# Clinical factors influencing serum CA125 levels and their implications for diagnosing ovarian tumors

**DOI:** 10.6026/973206300222481

**Published:** 2026-04-30

**Authors:** Neha Bharti, Nisha Jha, Sanjeev Kumar, Sude Singh

**Affiliations:** 1 Department of Biochemistry, ESIC Medical College & Hospital, Bihta, Patna, Bihar, India; 2 Department of Biochemistry, Netaji Subhas Medical College & Hospital, Bihta, Patna Bihar, India; 3 Department of Biochemistry, Darbhanga Medical College, Darbhanga, Bihar, India

**Keywords:** CA125, ovarian cancer, biomarker, benign and malignant tumors, diagnostic utility

## Abstract

Ovarian tumors remain a major gynaecological health problem and are among the leading causes of cancer-related mortality among women in India. Therefore, it is of interest to evaluate serum CA125 levels in women with ovarian tumors and examine their association with clinical diagnosis and risk factors such as age, body mass index (BMI), smoking, parity, and socioeconomic status. Hence, a total of 100 patients with ovarian tumors (50 benign and 50 malignant) and 50 age-matched healthy controls were included, and serum CA125 levels were measured using ELISA. CA125 levels were significantly higher in malignant tumors (1045.28 ± 281.96 U/ml) compared with benign tumors (186.48 ± 103.20 U/ml) and controls (18.50 ± 12.35 U/ml; p < 0.005), with significant associations observed with increasing age, higher BMI, smoking status, lower parity, and higher socioeconomic status. Thus, the combined diagnostic and risk-stratification value of CA125 with demographic and lifestyles factors help improve early detection and clinical assessment of ovarian tumors.

## Background:

Ovarian cancer is one of the most frequent and lethal gynecological malignancies affecting women worldwide, with a particularly high incidence and mortality rate in India population based registries generally report ASR values roughly in the 4-8 per 100,000 ranges, with urban registries such as Delhi and Pune at the higher end [1]. Ovarian cancer as an important but less common female malignancy globally, with an age standardized incidence around 6-7 per 100,000 women per year, consistent with GLOBOCAN 2020 estimates of about 314,000 new cases worldwide in 2020 [2]. It is often referred to as a "silent killer" due to the absence of specific early symptoms and the lack of effective screening tools for timely detection [3]. The disease largely arises from the ovarian surface epithelium, where chronic overstimulation by female hormones-such as estrogen, luteinizing hormone (LH) and follicle-stimulating hormone (FSH)-can cause epithelial erosion and neoplastic transformation [4]. Ovarian cancer is often asymptomatic in its early stages and thus most patients have widespread disease at the time of investigation [5]. Among various biomarkers, cancer antigen 125 (CA125), a high-molecular-weight glycoprotein derived from coelomic epithelium, is widely recognized as a tumor marker in epithelial ovarian carcinoma [5]. CA125 is expressed as a membrane bound protein at the surface of cells that undergo metaplastic differentiation into mullerian type epithelium or released in soluble form in bodily fluids [7]. It is normally expressed in tissues of Mullerian origin, including the endometrium, fallopian tubes and peritoneum, but not on the ovarian surface epithelium [8]. A huge number of novel biomarkers have been presented as promising in the diagnosis of ovarian cancer but CA125 is in the top and currently most used in clinical practice [9]. Elevated serum levels of CA125 are observed in approximately 80-95% of women with clinically detectable ovarian cancer, particularly in advanced stages [9]. The normal reference range for serum CA125 is considered below 35U/ml. Additionally, more people are reporting benign gynaecological diseases such endometriosis and pelvic inflammatory disease (PID) in part because of improved awareness and diagnosis [10]. An estimated 6-10% of women of reproductive age worldwide suffer with endometriosis, which is becoming more widely acknowledged as a significant contributor to infertility and persistent pelvic pain [11]. Benign disorders include endometriosis, PID, fibroids, pregnancy, menstruation and benign ovarian cysts can cause mild to moderate rise of CA-125 [9].

Although CA125 is not an effective screening tool for asymptomatic women due to limited specificity and false positives in benign conditions [6], it remains an important diagnostic aid in women at high risk of ovarian cancer such as those with a family history of hereditary ovarian malignancies when used in combination with pelvic ultrasonography and transvaginal sonography [12]. Because benign disease can also raise CA 125, a single value just above 35 U/mL has limited specificity; very high values (for example >200 U/mL) in a woman with an adnexal mass, especially postmenopausal, are much more suggestive of malignancy than benign pathology [13]. Age, body mass index (BMI), parity, smoking and socioeconomic status have also been identified as potential risk modifiers associated with altered CA125 levels and disease progression [9]. The present study was undertaken to measure serum CA125 levels in patients with ovarian tumors and to correlate these values with clinical diagnosis, demographic factors, and tumor type. By comparing patients with benign and malignant ovarian tumors to healthy controls, the study aims to evaluate the diagnostic significance of CA125 and its association with known risk factors, thereby enhancing its clinical interpretability in the Indian population. CA125 is considered an important predictive and prognostic biomarker in CA125-positive ovarian cancers and plays a significant role in the diagnosis, prognosis, and monitoring of treatment response in ovarian cancer patients. The protective effect of early and multiple pregnancies is believed to result from a reduced number of lifetime ovulatory cycles and hormonal fluctuations, whereas late marriage and delayed or absent childbearing prolong uninterrupted ovulation, which has been associated with an increased risk of ovarian carcinogenesis. Therefore, it is of interest to evaluate the diagnostic significance of serum CA125 levels and their association with clinical and demographic risk factors in patients with ovarian tumors.

## Materials and Methods:

## Study design and population:

The present cross-sectional comparative study was conducted to evaluate serum CA125 levels in patients diagnosed with ovarian tumors and to correlate their levels with clinical diagnosis and associated risk factors. For that total of 150 female subjects, aged above 20 years, in which 50 control group healthy women without any adnexal pathology, 50 benign ovarian tumors and 50 malignant ovarian tumors diagnosed with histopathological confirmed malignant ovarian tumors. All participants were enrolled from the department of Biochemistry, Darbhanga medical college.

## Sample collection:

A 2 ml of venous blood collected from the antecubital vein by syringe with all antiseptic precaution. The serum was separated and stored at -80°C for farther analysis. Serum CA125 levels were estimated using a through Enzyme Linked Immunosorbent Assay (ELISA) as per protocol. A detailed clinical history was obtained from each participant, covering age, parity, smoking habits, BMI and socioeconomic status.

## Statistical analysis:

All data were expressed as mean ± standard deviation (SD). Statistical analysis was performed using the student's t-test and one-way ANOVA, followed by appropriate post hoc tests to assess the significance of differences among groups. Correlations between serum CA125 levels and clinical parameters (age, BMI, smoking, parity and socioeconomic status) were evaluated using the Pearson correlation coefficient. A p < 0.05 was considered statistically significant.

## Results:

The present study included 150 female patients divided into three groups: 50 healthy controls, 50 patients with benign ovarian tumors and 50 patients with malignant ovarian tumors. All study participants were above 20 years of age. The clinical and demographic parameters studied were age, body mass index (BMI), smoking habits, parity and socioeconomic status and their correlation with serum CA125 levels was evaluated. The mean serum CA125 level in the control group was 18.50 ± 12.35 U/ml in the range of 2 - 44 U/ml, it was observed that in benign ovarian tumor group it was 186.48 ± 103.20 U/ml and in the malignant ovarian tumor group it was 1045.28 ± 281.96 U/ml. The difference between the three groups was statistically significant (p < 0.005) showed in Table 1 (see PDF). This finding indicates a progressive increase in serum CA125 concentration from normal to benign and malignant conditions. The age specific incidence rate (ASIR) for ovarian cancer revealed that the disease affects the women [14]. A significant inverse correlation was observed between age and serum CA125 levels among healthy controls (p = 0.001), indicating a gradual decline of CA125 levels with increasing age. In benign ovarian tumor cases, serum CA125 levels also decreased with age (p < 0.005) Figure 1 (see PDF). However, in malignant ovarian tumor patients, CA125 levels showed a positive correlation with age, increasing steadily in older age groups (p < 0.005).

Higher level of CA125 observed in obese women. Individuals with a BMI of 30Kg/m^2^ or higher have a 23 % higher risk of cancer than non-obese individuals [15]. The mean serum CA125 levels increased with rising BMI in all study groups. In the control group, the mean levels were 11.29 ± 6.98 U/ml for BMI < 25 kg/m^2^, 16.56 ± 11.31 U/ml for BMI 25-29 kg/m^2^ and 35.20 ± 17.6 U/ml for BMI ≥ 30 kg/m^2^ (p < 0.005). A similar trend was noted in malignant ovarian tumor cases where levels rose from 948.57 ± 308.28 U/ml (< 25 kg/m^2^) to 1099.41 ± 273.79 U/ml (> 30 kg/m^2^; p < 0.005) as showed in Figure 2 (see PDF). These findings demonstrate a positive association between obesity and elevated CA125 concentration. In the control group, there was no significant difference in CA125 levels between smokers (SM) (19.15 ± 11.53 U/ml) and non-smokers (NSM) (19.99 ± 11.61 U/ml; p = 0.654). However, in the benign and malignant groups, smokers had significantly higher CA125 concentrations (221.52 ± 113.92 U/ml and 1065.89 ± 265.27 U/ml, respectively; p < 0.005**) as showed in Figure 3 (see PDF), suggesting that smoking may influence tumor-related antigen expression. Serum CA125 levels declined with increasing parity across all study groups. Among healthy controls, mean values were 27.73 ± 9.55 U/ml (nulliparous), 22.53 ± 12.00 U/ml (primiparous) and 9.8 ± 7.94 U/ml (multiparous; p < 0.005). A similar trend was observed in malignant ovarian tumor patients, where mean levels were 1061.30 ± 255.87 U/ml, 1025.43 ± 243.23 U/ml and 998.93 ± 235.06 U/ml respectively (p < 0.005) as showed in Figure 4 (see PDF). These findings indicate that lower parity is a potential risk factor for elevated CA125 levels and malignant transformation. Serum CA125 levels showed a positive association with socioeconomic status. In the control group, mean CA125 levels were 270.16 ± 89.04 U/ml for high socioeconomic status, 161.00 ± 84.71 U/ml for middle and 130.33 ± 80.60 U/ml for low status (p < 0.005) as showed in Figure 5 (see PDF). A similar pattern was noted in benign and malignant groups, suggesting lifestyle and dietary factors associated with higher socioeconomic classes may contribute to increased CA125 expression. Direct comparison revealed that CA125 levels were substantially higher in malignant tumors (1045.28 ± 281.96 U/ml) compared to benign tumors (186.48 ± 103.20 U/ml; p < 0.005**) as showed in Figure 6 (see PDF). The increasing trend of serum CA125 levels with tumor stage and size further supports its diagnostic and prognostic value in ovarian malignancy. Serum CA125 levels were significantly higher in malignant ovarian tumors compared with benign tumors and healthy controls, indicating its diagnostic value. CA125 levels increased with age in malignant cases but decreased in controls and benign tumors. Higher BMI and smoking were associated with elevated CA125, while higher parity showed a protective effect. These findings support CA125 as a useful biomarker for ovarian cancer detection and risk assessment.

## Discussion:

CA125 is a useful diagnostic and prognostic marker that rises substantially from healthy controls to benign and then malignant ovarian tumors and in this study its levels also showed significant associations with age, BMI, smoking, parity and socioeconomic status [6]. The markedly higher values in malignant cases are consistent with reports that 80-90% of women with advanced epithelial ovarian cancer have CA125 levels above the conventional cut-off of 35 U/ml [16]. The findings reinforce that, while not disease-specific, CA125 remains the most widely used and clinically recommended tumor marker in the diagnosis and follow-up of ovarian cancer [17]. At the same time, the findings highlight that CA125 values are not static, but are significantly influenced by demographic, reproductive and lifestyle factors, which must be considered when interpreting results [18]. Age-related analysis revealed an inverse correlation between age and CA125 levels in healthy and benign groups, whereas a positive correlation was observed among malignant cases [19]. Conversely, the progressive rise with increasing age in malignant cases suggests that CA125 expression may be enhanced by age-related genomic instability, chronic inflammation, or delayed detection of advanced-stage tumors in older women [20]. The positive association between body mass index and CA125 in tumor groups suggests that obesity-related hormonal and inflammatory changes may amplify tumor marker expression and potentially reflect higher risk or more advanced disease [21]. Smoking had little or even negative association with CA125 in healthy women but showed a positive association in benign and malignant ovarian tumors, indicating that tobacco exposure may interact with tumor biology or antigen metabolism rather than simply raising baseline levels [22]. Low fertility, especially nulliparity, showed a strong association with higher CA125 in ovarian tumors, supporting the well-known link between reduced parity, higher lifetime ovulatory cycles and increased ovarian cancer risk [23]. Parity emerged as another key determinant, with CA125 levels decreasing progressively with increasing parity in all groups [24]. Nulliparous women had the highest CA125 values, while multiparous women demonstrated significantly lower concentrations [25]. Reduced parity is a well- established risk factor for ovarian cancer, possibly due to uninterrupted ovulatory cycles leading to repeated epithelial injury and inflammation [26]. Socioeconomic status (SES) also showed a positive association with serum CA125 levels. Women from higher socioeconomic backgrounds had greater CA125 values across all groups [27]. This pattern may reflect lifestyle factors, dietary habits, delayed childbearing or increased exposure to environmental risk contributors common in higher SES populations [28]. Finally, the direct comparison between benign and malignant tumors demonstrated a stark rise in CA125 levels in malignancy, affirming its diagnostic value [29]. The strong statistical association emphasizes the utility of CA125 not only for diagnosis but also for distinguishing benign from malignant ovarian masses [30]. Overall, this study reinforces CA125 as an easy, rapid and sensitive test with substantial diagnostic and prognostic utility but underscores that its optimal use requires contextual interpretation alongside age, BMI, smoking, fertility, socioeconomic factors and clinical-imaging findings rather than as an isolated screening tool. The risk of ovarian malignancy algorithm, which divides women with adnexal masses into low- or high-risk groups based on serum HE4 and CA-125, can achieve diagnostic accuracy of 90-95% in some trials.

## Competing interests:

The authors declare that they have no known competing financial interests or personal relationships that could have appeared to influence the work reported in this paper.

## Funding:

The authors received no funding.

## Availability of data statement:

Availability of data is not applicable as this study involves publicly available data.

## Consent for publication:

Consent for publication is not applicable as this study involves publicly available data.
